# Precision Military Medicine: Conducting a multi-site clinical utility study of genomic and lifestyle risk factors in the United States Air Force 

**DOI:** 10.1038/s41525-016-0004-1

**Published:** 2017-01-19

**Authors:** Susan K. Delaney, Ruth Brenner, Tara J. Schmidlen, Michael P. Dempsey, Kim E. London, Erynn S. Gordon, Mark Bellafante, Ashley Nasuti, Laura B. Scheinfeldt, Kaveri D. Rajula, Leo Jose, Joseph P. Jarvis, Norman P. Gerry, Michael F. Christman

**Affiliations:** 1 Coriell Institute for Medical Research, 403 Haddon Avenue, Camden, NJ 08103 USA; 2 Air Force Medical Support Agency, Defense Health Headquarters, Falls Church, VA 22042 USA; 3 Air Force Research Laboratory, Institutional Review Board, 711 HPW/IR, Wright-Patterson AFB, OH 45433 USA; 4 Present Address: Genomics Medicine Ireland, Cherrywood Business Park, Co. Dublin, Ireland; 5 Present Address: Genome Medical, Inc., 222 Amberfield Drive, Mount Laurel, NJ 08054 USA; 6 Present Address: Institute for Genomics and Evolutionary Medicine, Temple University, SERC Building 1925 N. 12th Street, Philadelphia, PA 19122 USA; 7 Present Address: Advanced BioMedical Laboratories, 1605 Industrial Highway, Cinnaminson, NJ 08077 USA

## Abstract

Following several years enrolling disease-specific and otherwise healthy cohorts into the Coriell Personalized Medicine Collaborative, a prospective study aimed at evaluating the clinical utility of personal genomic information for common complex disease and pharmacogenomics, the Coriell Personalized Medicine Collaborative expanded to create a military cohort, specifically, the United States Air Force. Initial recruitment focused on Air Force Medical Service personnel and later expanded to include all Active Duty Air Force members and beneficiaries. Now in its 6th year, the study has produced a wide variety of insights, including optimal study design for military-sponsored genomic research, and discussion on genetic information sharing between and amongst Air Force study participants, civilian and military researchers, and the United States Department of Defense. Over the longer term, analyses will further contribute to the development of policies and processes relevant to clinical decision support and data sharing within the US military, and on-going work with the Air Force Medical Service sub-cohort will generate critical insights into how best to deploy useful genomic information in clinical care. Here we discuss challenges faced and critical success factors for military-civilian collaborations around genomic research.

## Introduction

In 2010 the Air Force Medical Service (AFMS) Patient-Centered Precision Care (PC2-Z) Program was established as an Air Force Surgeon General Directive and launched by the AFMS Innovations to gather clinical knowledge and provide recommendations for translating genome-informed medicine into precision healthcare for all Air Force (AF) healthcare beneficiaries. Recognizing its established infrastructure, the Coriell Personalized Medicine Collaborative (CPMC) was chosen as the centerpiece of the PC2-Z Program as a clinical utility study (CUS). The CPMC is an ongoing prospective study investigating the impact of personalized genetic risk reports for common complex diseases and drug metabolism on health behavior and outcomes; the CPMC’s actionable variant list is available on the study website.^1^ An overview of the CPMC study,^2^ the CPMC’s approach to genetic risk estimation for health conditions,^3^ the study’s pharmacogenomics appraisal, evidence scoring and interpretation system,^4^ consenting and participant interaction model,^5^ early findings in clinical utility and informing genetic counseling practices,^6–11^ and new genetic associations^12^ have been described elsewhere. Given the CPMC’s agile study design, only minor modifications were required to accommodate the CUS objective to establish best practices in the implementation of precision military medicine.

As a new AF initiative aimed at enhancing Air Force Medicine, AFMS Innovations chose to focus recruitment for Phase I of the study on AFMS personnel. Hypothesizing that AFMS personnel will be better prepared to deal with genomic information in the clinic if they have had personal, first-hand, experience with their own genomic information, a concept that has since been tested and supported by others.^13–15^ Phase I of the CUS (2012–2014) enrolled 2103 Active Duty and civilian members from the AFMS, exceeding the enrollment goal of 2000. The recruitment of AFMS-only personnel in Phase I served three purposes: (1) to disseminate the concept of genomic medicine to and amongst Air Force healthcare providers; (2) to educate and prepare AFMS personnel for the growing incorporation of genomics in clinical care; and (3) to pilot test the assumptions that members of AFMS population would better understand genetics concepts, would react appropriately to genetic risk information and/or would be less fearful of genetic information compared to their peers in non-medical career fields.

By early 2015, the desire to enlarge and diversify the cohort for more powerful analyses called for an expansion of the eligibility criteria to include all Air Force Active Duty members and their spouses, as well as Air Force retirees and their spouses. Table 1 describes the current eligibility criteria for the CUS cohort. As of August 2016, more than 3140 participants have been enrolled in the CUS; the total enrollment goal is 6500 by late 2017. Table 2 describes the CUS cohort as of August 2016.

**Table 1:** CPMC Air Force Clinical Utility Study, current eligibility criteria

1. 18 years of age or older
2. Possession of a valid personal email address^a^
3. Current active-duty member of the United States Air Force or spouse of active-duty member
4. Retiree of the United States Air Force or spouse of retiree
5. Possession of a DoD medical record (current Armed Forces Health Longitudinal Technology Application (AHLTA)
6. Defense Enrollment Eligibility Reporting System (DEERS) eligible
7. Currently enrolled and seeking care at a United States Military Treatment Facility (MTF)
8. Enrolled in *TRICARE Prime* health care program of the United States Department of Defense Military Health System^b^

^a^Air Force subjects are asked to provide a personal email address rather than their military-issued email address. This reduces possible access to a participant’s study data as communications using, or data stored on, U.S. Government Information Systems are not private and are subject to monitoring, interception, and search.

^b^Restriction to TRICARE Prime enrollment vs. other TRICARE health plans, e.g., TRICARE For Life, is relevant to accessibility to subjects’ electronic medical record; also, non-Prime beneficiaries often see providers outside the MTF and therefore medical visits are not always documented in a standardized format within AHLTA.

**Table 2:** CPMC Air Force Clinical Utility Study, demographics

	Phase 1	Phase 2
	2012–2014	Jan 1 2015 to Aug 31 2016
	*N* = 1325 (%)	*N* = 556 (%)
Age		
18–24	0.91	7.55
25–34	27.40	36.33
35–44	31.62	32.91
45–44	25.96	17.09
55–64	12.83	5.22
65–74	1.28	0.90
Gender		
Male	49.13	51.26
Female	50.87	48.74
Ethnicity		
African American	8.08	8.09
Asian	3.55	4.14
Caucasian	79.92	77.52
Hawaiian/Pacific Islander	0.60	0.18
Mixed race	6.04	6.83
Native American	0.30	0.36
Did not want to answer	1.51	2.88

## Charting a new course in military precision medicine research

The CUS protocol is the first USAF research study to return genome-based risk reports to healthy participants. Charting this novel course required more specific considerations of military human subject research such as presence of superiors during recruitment activities (perceived coercion), Cadet/Basic Trainee enrollment in a “Greater than Minimal Risk” (GTMR) protocol, genetic privacy protections, and national security of personnel medical and deployment data.

The protocol was presented to the Air Force Research Laboratory (AFRL)/Institutional Review Board (IRB) at Wright-Patterson Air Force Base, Ohio; the protocol was approved in January 2012 as a multi-site protocol, thereby reducing potential regulatory burden via required approvals at each recruitment site. The Coriell Institute for Medical Research IRB defers approval to the AFRL IRB to ensure “local context” based on AFRL’s institutional knowledge of military research regulations, policies, et cetera.

Due to the initial classification of the protocol as GTMR additional review and approval concurrence by the higher headquarters/AF Component office of human research compliance oversight (AFMSA/SGE-C) was also required. The GTMR determination was initially based on concerns related to (1) privacy—who would have access to data and how that could affect the military career or benefits of participants?; (2) protection from discrimination—Active Duty military are excluded from GINA;^16^ (3) risk of coercion/perceived coercion—due to the top down structure of the military there is unique sensitivity to coercion with respect to human subject research; (4) impacts on subjects with the return of genetic testing results. In addition, research within this unique setting posed additional considerations including whether participants could continue in the study if they separated from the military during the study period and who would cover the cost of study related injuries if they occurred (military or civilian medical services).

Despite initial concerns that warranted a GTMR determination, in February 2016 that determination was reduced to minimal risk. This change in risk determination was based on evidence submitted by the CPMC research team coupled with consultative inputs to and observations by the AFRL board. To begin, the privacy and GINA exclusion concerns were addressed with critical assessment of multiple GINA-like privacy and strong non-discrimination protections embedded within military/Air Force regulations. Secondly, coercion concerns where mitigated with development of strict on-site recruitment procedures. The direct return of results risk impact was re-addressed with additional evidence. Survey data provided by study subjects after viewing ten complex disease reports demonstrated that more than 80% of participants self-reported either low anxiety or no heightened anxiety after receiving test results. Additionally, a low number of CUS subjects seeking genetic counseling was observed. This finding was further supported by a 2014 paper authored by the CPMC study team^8^ which described a retrospective qualitative review of notes from 157 genetic counseling inquiries received by CPMC genetic counselors from the CPMC non-military cohorts (*n* = 2636) between April 1st 2009 and April 30th 2011 and determined that CPMC participants were mostly comfortable interpreting genomic test results on their own and were not overwhelmed by multiplex testing.

### Novel recruitment methodology

**Fig. 1:**
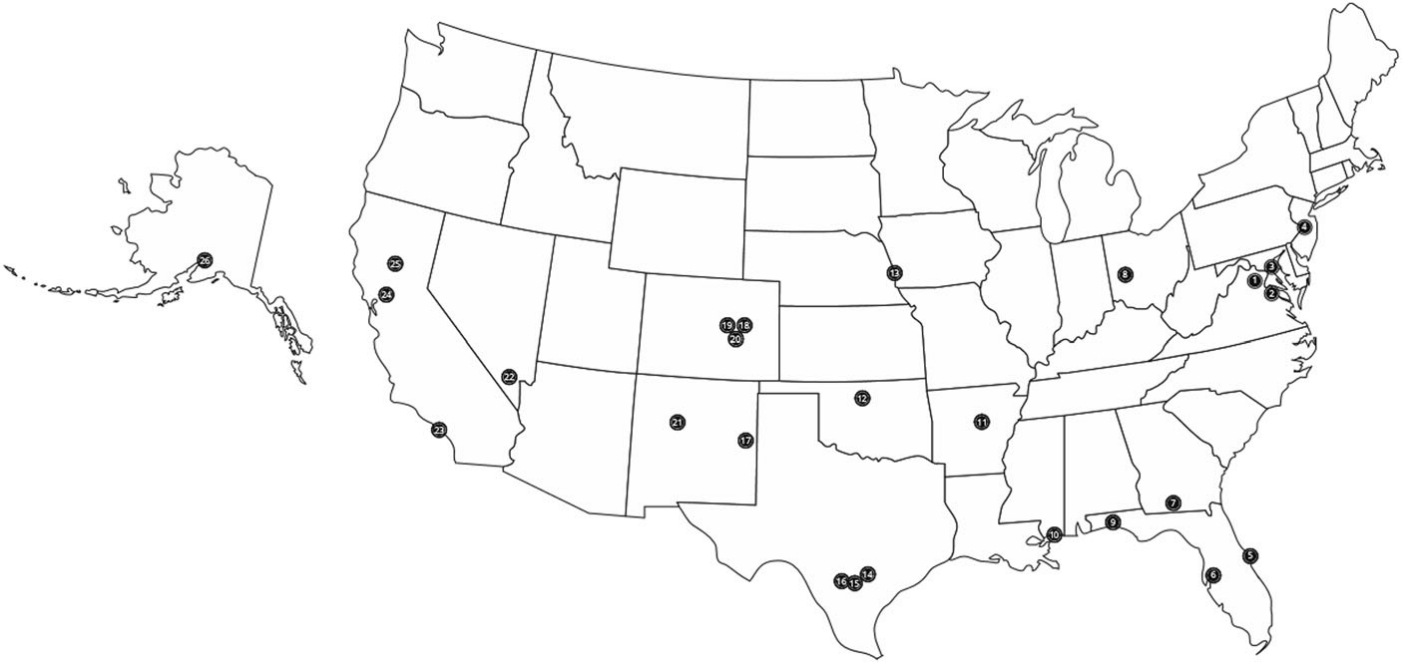
*1* DHHQ, Falls Church, VA; *2* Andrews AFB; *3* Bolling AFB; *4* JBMDL; *5* Patrick AFB; *6* MacDill AFB; *7* Moody AFB; *8* Wright Patterson AFB; *9* Eglin AFB; *10* Keesler AFB; *11* Little Rock AFB; *12* Vance AFB; *13* Offutt AFB; *14* Randolph AFB; *15* Air Force Medical Operations Agency; *16* Lackland AFB; *17* Cannon AFB; *18* Schriever AFB; *19* Peterson AFB; *20* United States Air Force Academy; *21* Kirtland AFB; *22* Nellis AFB; *23* Los Angeles AFB; *24* Travis AFB; *25* Beale AFB; *26* Elmendorf AFB

Equipped with a multi-site protocol, the Coriell CUS research team has conducted on-site recruitment at more than 20 military installations across the United States (Fig. 1); at times, recruitment events were held on multiple occasions at a given base.

Phase I enrollment centered at Military Treatment Facilities and clinical sites, (e.g., 81st Medical Group at Keesler AFB). Approval for recruitment at each Medical Group was provided by the respective Medical Group Commander. When eligibility broadened to all Active Duty personnel, recruitment activities also broadened to non-medical locations such as fitness centers and other base common places, and approval from the Base Commander became a requirement. Requests for these approvals are originated by the AFMS Innovations Chief of Personalized Medicine and include an outline of the regulatory requirements for conducting research in the military. An important aspect of these regulations includes special attention to the role of leadership at research recruitment events and the need to avoid any perception of coercion, as codified in military regulations.^17^ To reduce the risk of coercion or perceived coercion enrollment events were conducted by non-military personnel employed by Coriell. Study staff made an announcement prior to the beginning of the group enrollment session reminding attendees of DoD Instruction 3216.02, which establishes policy and assigns responsibilities for the protection of human subjects in DoD-supported programs and requesting that all superiors of service members talk with study staff about private enrollment opportunities to avoid any appearance of coercion. In addition, approved ombudsmen were present at all group enrollment sessions as observers to ensure that the research study was being presented and conducted in an ethical manner.

Planning a CUS recruitment event begins several weeks in advance of the planned date(s). Coriell engages base-specific points-of-contact (POCs) to coordinate logistics from inside the base perimeter. Weekly communications with the POC in the 6–8 weeks prior to recruiting events ensures potentially interested volunteer participants are aware that the Coriell study team will be on base on a scheduled date(s). POCs are typically eager to engage in research planning as an important part of their own professional education and advancement.

Recruitment and enrollment takes approximately 30–40 min per volunteer participant. After expressing interest to Coriell study staff potential participants view an AFRL/IRB-approved 20-min video on an iPad which describes the study and purpose, what to expect as a CUS participant, the risks and benefits of participation, what they may learn during their participation, who to contact if they need to report an adverse event, and other key details about the study. This video enables a comprehensive and consistent review of the informed consent document (ICD) and enables the efficient enrollment of multiple participants at once and at staggered times. It is intended to supplement the ICD which is reviewed and signed after the video presentation. The CUS is a complex endeavor and ongoing participation—up to 10 years—is a significant commitment. Participants have been observed to be comfortable sitting at the iPad table in groups of four or more to watch the video.

Potential enrollees are encouraged to ask questions regarding participation after watching the video; approximately 10% of participants pose additional questions and common queries include the definition of actionable vs. non-actionable conditions; access to results outside the scope of the study, e.g., allergies and Alzheimer’s disease, or the impact of results on biological children. After completing the required paperwork, including the ICD and information privacy (HIPAA) documents, the enrolled participant provides a small (2 ml) saliva sample.

The on-base location of recruitment events is designed to reach as many potential participants as possible during their working (duty) hours. The recruitment occurs in high-traffic areas, e.g., pharmacy waiting areas, medical and dental clinic entrances, and fitness centers. The presence of the study staff, a table with CUS enrollment supplies (saliva collection kits, iPads, and regulatory paperwork) and posters welcome personnel to enquire about the CUS. In the weeks leading up to recruitment events, a variety of AFRL/IRB-approved recruitment materials are used, including posters and social media posts. Of note, in addition to IRB approval, recruitment communications must be agreed to by the base or MTF commander. Careful consideration is paid to whom the message comes from (communications office vs. base commander) to avoid any perception of coercion to participate. Approximately 95% of USAF CUS participants have enrolled at in-person recruitment events with the remaining number enrolling online. The remote recruitment in the CUS study was approved as a reasonable option (combined with on-site events) to provide for an equitable opportunity for all USAF service members to participate privately.

### CUS participant engagement

The CUS study design requires consented participants to activate their secure CPMC web portal account which is the primary conduit by which they receive their personalized risk reports and provide survey data back to the CPMC research team. As of August 2016, 69% (3321/2293) of enrolled CUS subjects activated their CPMC account. Of those CUS subjects who have activated their account, 82% (2293/1881) (56% of those consented) completed their baseline survey questionnaires (medical history, family history, lifestyle, etc.); this rate exceeds that typically observed in survey of medical professionals survey response rates are typically low among medical professionals with similar research studies reporting survey response rates among medical professionals between (20–44%).^16–18^

With all participatory study designs, some degree of inactivity and disengagement is expected. The research team regularly examines engagement within the USAF cohort. In late 2013 we reviewed our progress across then-completed on-base enrollment events (*n* = 1957 individuals): 15 out of 18 bases produced account activation rates greater than 60% and nearly half of these bases (8) show rates greater than 75%. In addition, there was no striking trend within those 30% who did not activate their accounts following recruitment with respect to base location or size, subject age or gender, or at which time point during the study they enrolled.

Participants receive at least one report per month after activating their account, completing the required baseline questionnaires, and successful genotyping of their sample. The number of reports accessible to any participant therefore varies based on their time since enrollment, however, at maximum, 36 reports are available to participants. 96% of AF cohort participants have viewed at least one report; specifically, 61% AF personnel who completed their baseline questionnaires have viewed at least one half of their available reports. Close to 43% AF cohort participants have viewed three quarters or more of their available reports and 8% have viewed all available reports. These numbers suggest robust and ongoing participant engagement with the project. Again, no apparent differences in report viewing were observed across base sites, suggesting the current engagement provides accurate insights into the expected rates of activation, baseline completion, and report viewing within the proposed sample size of 6500.

## Discussion

The CPMC-Air Force CUS has revealed several best practices for the implementation of a service-wide CUS. We present them here as five “critical success factors”:

### A committed vision

As with any study, a committed vision is necessary. In the case of a civilian-military partnership where each group is accustomed to different rules of conduct, ensuring that both parties are aligned and committed to the same vision is essential. With this commitment, researchers should not be hesitant, within appropriate parameters, to test the boundaries of regulatory pathways and standard approaches to accomplish increasingly novel studies within the military context.

### Collaborative approach to regulatory oversight

The CUS study benefitted enormously from a highly-accessible collaboration between Coriell Institute and AFRL. It has proven to be a best practice for the civilian collaborator (Coriell) to maintain continual regulatory consultation because (a) there are unique military settings that must be considered; (b) there are unique military regulations that a civilian institution may not be adequately-versed; and (c) there was, in many cases, no precedent for the application of existing regulations to a study of this type, lending itself to setting precedent at times. The success of this effort can be attributed to AFRL’s commitment to working collaboratively to guide the study design within the confines of the military human subject regulations with Coriell contributing expertise, insights, and comparisons to this area in the civilian sector.

### Understanding privacy in military research

Privacy is a fundamental topic in biomedical research and it was particularly present in the context of a study that returned genetic risk reports to Active Duty Air Force personnel. Among other segments of the US government, the Genetic Information Nondiscrimination Act (2008) does not apply to the Active-Duty US military members. Protection of Air Force research subjects is well-established by current DoD and USAF policies and procedures. Air Force Instruction 36–2706, Equal Opportunity Program, (1.1.1.) prohibits “… any Airman, military or civilian, to unlawfully discriminate against, harass, intimidate or threaten another Airman on the basis of race, color, religion, sex, national origin, age, disability, reprisal, or genetic information.”^18^ Additionally, Air Force Instruction 48–123 and the Air Force Medical Standards Directory identifies disqualifying medical conditions for enlistment and retention.^19^ Identification of a listed medical problem directs medical providers to request a waiver for the Service Member to proceed with induction or retention in the USAF. These documents do not identify genetic markers/genotype as disqualifying for service, although phenotype may be depending on the condition. Candidates may be asked about family history as part of a complete medical exam, however they do not undergo genetic testing for this prior to enlistment. For example, if a candidate has a family history of breast/ovarian cancer, or if a candidate (or Service Member) is known to be BRCA+, they will not be disqualified based on existing standards. However, expressing the gene—i.e., having breast cancer (whether related to genetic mutation or not)—is disqualifying. It is important to note that the purpose of “disqualifying” a person is not to withdraw them from service; rather it is to provide them with a designation that may prevent deployment, recognition of the need for treatment/monitoring for a specified amount of time at a major medical center, determine need for advanced care, etc. This kind of nuanced understanding came from detailed conversations between Coriell and Air Force and provided for an accurate assessment of the matter that subsequently informed the protocol, the ICD, and Coriell study staff for discussions with potential CUS enrollees.

### Boots on the ground

The CUS study design could have called for online/remote enrollment only or utilized a network of military physician-researchers to conduct enrollment. Instead, AFMS recognized both the short-term and long-term benefits of facilitating on-base recruitment activities executed by non-military personnel with expertise in clinical genetics research. In the short-term, the Coriell team was better equipped to describe the study to potential enrollees, provide comprehensive informed consent, and then ensure strong chain-of-custody practices for transporting and accessioning subject samples and documentation, while reducing the risk of coercion from peers or commanding officers. Longer-term, the cross-base experience of the Coriell team led to a deep understanding of military installation dynamics and evolved recruitment events accordingly to maximize participant engagement. Meaningful conversations with potential and current CUS participants provided valuable insights which were then incorporated into enrollment materials, identification of downstream resources such as report educational materials, and recommendations to AFMS leadership. In summary, we recommend that civilian researchers pursuing similar military engagements make every effort to have a physical presence at any engaged military facility throughout the recruitment, and if appropriate study execution, to fully appreciate the nuances of a sector that differs in many ways to the traditional academic medical research setting.

### Engaging motivated and skilled points-of-contact

Undoubtedly, the success of the CUS to date can be traced in large part to the more than forty points-of-contact (POCs)﻿ across Air Force installations and sites. These motivated POCs proved extraordinarily important in securing base approvals, identifying key locations for recruitment activities, liaising with leadership, and communicating the CUS presence and availability. From seemingly simple issues like physical access to the base which must be facilitated by military personnel to institutional knowledge and insider perspective regarding perception of research at individual bases, ideal recruitment locations, etc., civilian researchers are not likely to be successful without engaging effective POCs.

## Conclusion

Multi-site studies always present a challenge in ensuring that leadership and personnel, as well as IRBs, across all sites are aligned. Likewise, and of paramount importance, is ensuring that potential participants are well informed and the goals of the study resonate with the local population. Here we present some unique challenges and opportunities for success when conducting civilian led multi-site research within the military and other special populations. As described, the success of the CPMC Air Force CUS to date has been based on mutual collaboration and commitment and the recognition of differences between participating organizations. Though it may be easy to identify similarities and diminish those differences, by recognizing the cultural and regulatory differences between military and civilian practices, this basic recognition allows both parties to engage more actively as true partners. For the genomics research field, particularly as large cohort studies are established, we hope the perspectives presented here are considered for more distinct research populations.
